# Ceramide kinase mediates intrinsic resistance and inferior response to chemotherapy in triple‐negative breast cancer by upregulating Ras/ERK and PI3K/Akt pathways

**DOI:** 10.1186/s12935-020-01735-5

**Published:** 2021-01-11

**Authors:** Shan Zhu, Yulin Xu, Lijun Wang, Shichong Liao, Yuan Wang, Manman Shi, Yi Tu, Yurong Zhou, Wen Wei

**Affiliations:** 1 Department of Breast and Thyroid Surgery, Renmin Hospital of Wuhan University, Wuhan, 430060 Hubei China; 2 Department of Radiology, Zhongnan Hospital of Wuhan University, Wuhan, 430071 Hubei China

**Keywords:** CERK, CP-1, TNBC, Chemoresistance, Ras, Biomarker

## Abstract

**Background:**

Clinical management of triple-negative breast cancer (TNBC) patients remain challenging because of the development of chemo-resistance. Identification of biomarkers for risk stratification of chemo-resistance and therapeutic decision-making to overcome such resistance is thus necessary.

**Methods:**

Retrospective analysis was performed to identify potential stratification biomarkers. The levels of ceramide kinase (CERK) was determined in breast cancer patients. The roles of CERK and its downstream signaling pathways were analysed using cellular and biochemical assays.

**Results:**

CERK upregulation was identified as a biomarker for chemotherapeutic response in TNBC. A > 2-fold change in CERK (from tumor)/CERK (from normal counterpart) was significantly associated with chemo-resistance (OR = 2.66, 95% CI 1.18–7.34), P = 0.04. CERK overexpression was sufficient to promote TNBC growth and migration, and confer chemo-resistance in TNBC cell lines, although this resistance could be overcome via CERK inhibition. Mechanistic studies suggest that CERK mediates intrinsic resistance and inferior response to chemotherapy in TNBC by regulating multiple oncogenic pathways such as Ras/ERK, PI3K/Akt/mTOR, and RhoA.

**Conclusions:**

Our work provides an explanation for the heterogeneity of chemo-response across TNBC patients and demonstrates that CERK inhibition offers a therapeutic strategy to overcome treatment resistance.

## Background

Breast cancer is the most common cancer among women, and remains a major health problem [1]. Based on the molecular expression of cell-surface receptors, breast cancer is classified into four subtypes: normal-like, estrogen receptor (ER)-positive (luminal), epidermal growth factor receptor 2-(HER2-) enriched, and basal like [2]. Triple-negative breast cancer (TNBC), which is characterized by of the absence of expression of HER2, ER, and progesterone receptor (PR), is the most aggressive subtype and does not benefit from hormonal or targeted therapies [3]. The only available clinical treatment option for TNBC, both early and advanced, is chemotherapy. However, the majority of patients rapidly develop resistance and undergo relapse [4]. The poor outcome and lack of therapeutic targets necessitate the identification of novel biomarkers for chemo-resistance risk stratification and therapeutic management.

Ceramide kinase (CERK) is a lipid kinase that regulates the level of ceramide and ceramide 1-phosphate (C1P) by phosphorylating ceramide to produce C1P [5]. Ceramide is pro-apoptotic and anti-proliferative in many tumor cell types, whereas C1P exerts the opposite effects as ceramide [6–10]. C1P is a bioactive sphingolipid, and plays critical roles in pathophysiology of cancer and inflammation [6]. As the only enzyme known to generate C1P, CERK has also been implicated in cancer cell growth, survival, inflammation, and dissemination [11]. Various studies have reported the functional role of CERK in breast cancer progression and recurrence. CERK promotes tumor cell survival and mammary tumor recurrence [12]. It is upregulated in metastatic breast cancer cells and contributes to migration and invasion by activating PI3K and Akt [13]. High CERK expression is associated with poorer prognosis in ER-negative breast cancer [14]. The association of CERK expression with poor clinical outcome is not limited to ER-negative breast cancer, but also found in other aggressive subtypes of breast cancer, including HER2+, basal-like, or high-grade breast cancer [12].

In this study, we demonstrate that CERK upregulation results in the activation of multiple oncogenic pathways in TNBC cells, and increased growth and migration. In addition, CERK upregulation has a profound effect on chemo-sensitivity and can serve as a biomarker for risk stratification for chemo-resistance in newly diagnosed TNBC patients.

## Materials and methods

### Cell culture, transfection, drugs, and antibodies

Three human TNBC cell lines were obtained from the Cell Bank of the Chinese Scientific Academy, and their identity was confirmed via human 9-Marker STR DNA profile analysis. Cell were cultured in Minimal Essential Media (MEM) containing 10% fetal bovine serum (FBS, Hyclone) and 2 mM L-glutamine (Invitrogen). Cells were seeded in a six-well plate at a density of 10^5^ cells per well. On reaching 80% confluence, cells were transfected with siRNA or overexpression plasmid using Dharmafect 1 (Dharmacon) by incubating Dharmafect 1 and siRNA or plasmid. siRNA against CERK: UAC AGG CAC AGA GCA GGC A (CERK siRNA#1) and UGC CUG CUC UGU GCC UGU A (CERK siRNA#2) and negative control siRNA (ON-TARGETplus Non-targeting siRNA) were purchased from Dharmacon. CERK was cloned into pCMV3-C-his vector (Sino Biological). Protein expression analysis was performed at 48 h post-transfection. Paclitaxel, cisplatin, LY294002, PP242 and rapamycin were from Selleck, and 10-DEBC was from R&D Systems.

### Patients and tissue specimens

This study was approved by the ethics committee of Renmin Hospital of Wuhan University. One hundred TNBC patients seen at the Renmin Hospital of Wuhan University and Zhongnan Hospital of Wuhan University between 2016 and 2019 were included in the study. None of the included patients had received treatment at the time of diagnosis. TNBC tissues and their adjacent normal breast tissues were obtained from TNBC patients at diagnosis, stored in − 80 °C, and used for mRNA and protein extraction.

### ELISA assays

Snap-frozen tissues were homogenized using a polytron homogenizer and lysed with ice-cold RIPA buffer (Invitrogen). Ceramide kinase level was determined using the Human Ceramide Kinase (CERK) ELISA Kit (Aviva Systems Biology). Cellular Ras, RhoA and Rac1 activity were assessed by Ras, RhoA, and Rac1 G-LISA Activation Assay Kit (Cytoskeleton Inc. US) using cell lysates.

### Proliferation assay

Cells were seeded in a 96-well plate at a density of at 10^4^ cells per well. After three days of drug treatment, cell proliferation was determined using BrdU Cell Proliferation Assay Kit (Abcam) according to the manufacturer’s protocol.

### Annexin V quantification

Cells were seeded in a six-well plate at a density of at 5 × 10^5^ cells per well. After three days of paclitaxel or cisplatin treatment, cells were detached using trypsin and resuspended in PBS. Cells were stained using the Annexin V-FITC kit (Beckman Coulter) in accordance with the manufacturer’s instructions. The quantification of Annexin V was performed using flow cytometry (MACSQuant).

### Boyden chamber migration assay

Cell migration assay was performed using a Boyden chamber (6.5 µm diameter, 8 µm pore size, 24 well-plate; Cell Biolabs). Transfected cells were incubated with serum-free MEM (starvation medium) for six hours and seeded into a transwell filter in starvation medium. MEM containing 10% FBS was placed into the lower chamber, and cells were allowed to migrate for 24 h. Unmigrated cells on the upper surface of the transwell filter were removed with a cotton swab. Migratory cells in the lower chamber were stained with 0.4% Giemsa. Migrated cells were counted in five different random fields per well under a light microscope.

### Western blot

Total protein was extracted from cells using RIPA buffer. Protein concentration was measured using the BCA protein assay kit (Abcam). Equal amounts of proteins were loaded onto SDS-PAGE gel, resolved by electrophoresis, and transferred to a PVDF membrane. Western blot was performed using standard protocol [15]. Antibodies used for western blot analysis were as follows: p-Akt(S473), p-ERK(T202/T204), p-mTOR(S2481), p-S6 (S235/236), p-PI3K(T458), p-MYPT1 (T853), and p-MLC(S19), and their corresponding total were from Cell Signaling. The protein bands on the membrane were visualized using the Western Lightning chemiluminescence reagent (PerkinElmer).

### Ceramide measurement

Cells were lysed for lipid extraction, and then mixed with lipid internal standard mix (Avant Polar Lipids). After centrifugation, the supernatant was removed; then, the pellet was dried and reconstituted in methanol. The levels of ceramide species were determined with a liquid chromatography mass spectrometry (LC-MS)/MS system utilizing an Agilent 1100 HPLC system coupled to an Applied Biosystems Sciex 4000 QTrap mass spectrometer, according to previously described procedures [16]. Quantification was performed using Mass Hunter Software (Agilent Technologies).

### RNA extraction and real time PCR

Total RNAs were extracted using TRIzol Reagent (Ambion) using a standard protocol. RNA was reverse-transcribed using Superscript III First-Strand Synthesis System (Invitrogen) and quantitatively assessed using the iQ5 Multicolor Real-Time Detection System (Bio-Rad) according to the manufacturer’s protocol. The sequences of the primers (BioRad) were F: 5′-GTC CTT CCT CCC AGC ACA G-3′ and R: 5′-GCA CTT CCG GAT AAG GAT GA-3′. The relative *CERK* mRNA expression levels were normalized to *GAPDH* levels.

### Statistical analyses

Data are expressed as mean and standard deviation (SD) to indicate data variability. Statistical analyses were performed by unpaired Student’s t test for cell assays. To evaluate the association of CERK (Tumor/Normal) ratio value with clinical resistance to chemotherapy in TNBC patients, a Kaplan Meier Estimate between different variables was calculated. Odds ratio (OR) was tabulated to demonstrate clinical significance. The statistical analysis for OR and 95% Confidence Interval (CI) were calculated using the Prism Software (GraphPad). P-values < 0.05 were considered to indicate statistical significance.

## Results

### CERK overexpression as a biomarker for chemotherapeutic response in TNBC

**Fig. 1:**
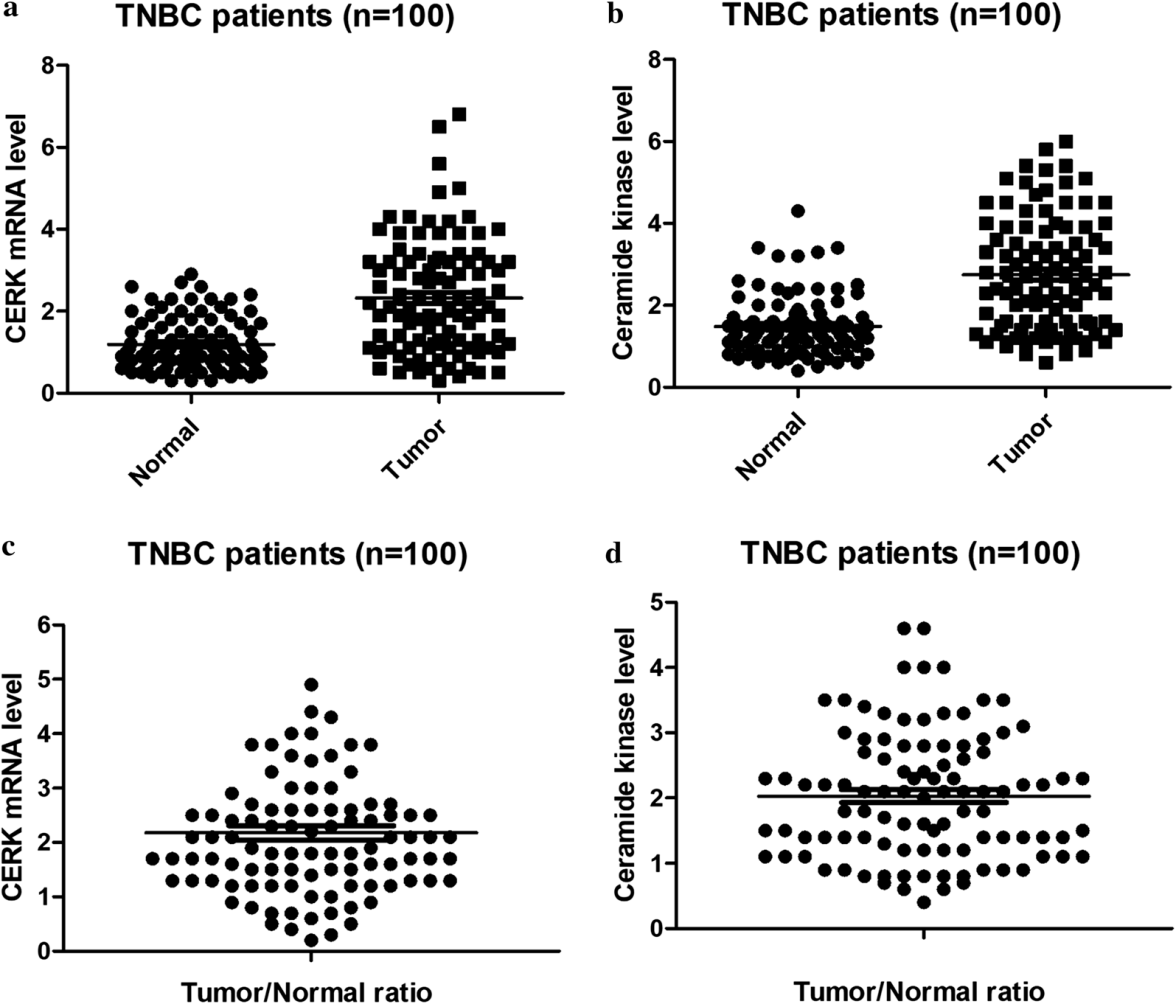
CERK is upregulated in TNBC patients. Scatter plot of CERK mRNA (**a**) and protein (**b**) in paired TNBC malignant tissues and adjacent normal tissues. Samples were obtained from 100 TNBC patients. The line shown in the scatter plot indicates the average expression of CERK in TNBC malignant and normal tissue. Average of CERK mRNA (**c**) and protein (**d**) ratios in the tumor and normal tissues of TNBC patients; *P < 0.05, compared with that in normal tissues

We first investigated the expression of CERK in paired TNBC tumor and adjacent normal breast tissue obtained from one hundred TNBC patients who has not received treatment as of yet. All patients included in this study were confirmed to have TNBC and given chemotherapy as the first-line treatment. We showed that the *CERK* mRNA level varied remarkably in both TNBC tumor and normal breast tissue, with the average higher in tumor tissue than in normal tissue (Fig. 1a). Consistent with this result, a similar phenomenon was observed for CERK protein levels in TNBC tumor and normal tissues, as shown by ELISA (Fig. 1b). The average level of CERK mRNA and protein in TNBC tumor tissue was approximately two-fold higher than normal (Fig. 1a, b). The ratio of CERK mRNA and protein levels in tumor and paired normal tissues from each TNBC patient was calculated and shown in Fig. 1c, d. The results demonstrated that total ~ 20% of TNBC tissue samples had a tumor/normal ratio of < 1 or > 3 and ~ 80% of TNBC tissue samples had a ratio of ~ 2, suggesting that CERK is upregulated in most of the TNBC patients.

We next performed a retrospective analysis of the influence of CERK tumor/normal ratio on chemotherapeutic response in TNBC patients. In the group of patients described above, we compared the clinical responses to first-line chemotherapy with anthracyclines (e.g., doxorubicin) and taxanes (e.g., paclitaxel) in individuals with CERK tumor/normal ratios of > 2 and < 2. We classified the clinical responses at six months after chemotherapy initialization according to the Response Evaluation Criteria in Solid Tumors (RECIST) version 1.1, which includes the classifications “complete response (CR)”, “partial response (PR)”, “stable disease (SD)”, and “progressive disease (PD)” [17]. We defined resistant individuals as those who under the SD and PD categories, and sensitive individuals as those who have achieved CR or PR. We found that patients with CERK ratio > 2 were more likely to have resistant disease than sensitive disease compared with those with CERK ratio < 2 (Table 1). The overall odds ratio (OR) for resistant disease among patients with CERK ratio > 2 compared with those with < 2 was 2.66 (P = 0.04, 95% CI 1.18–7.34), demonstrating that CERK is a biomarker for chemotherapeutic response in TNBC.

**Table 1:** Association of CERK upregulation with clinical resistance to chemotherapy in TNBC patients

CERK (tumor)/CERK (normal) in TNBC			
TNBC (patients) (n = 100)	< 2% (n)	> 2% (n)	
Resistance	43% (20)	75% (40)	OR = 2.66 95% CI (1.18–7.34) P = 0.04
Sensitive	57% (27)	25% (13)	

Subjects with newly diagnosed TNBC were divided into two groups: those with CERK(Tumor)/(Normal) > 2 and those with CERK(Tumor)/(Normal) < 2. Individuals were then classified as resistant (SD and PD as per RECIST version 1.1 criteria) or sensitive (CR and PR per RECIST version 1.1 criteria). Statistical analysis testing for the association between the CERK upregulation and clinical resistance to chemotherapy was carried out using the Kaplan Meier estimator. Odds ratio (OR) was tabulated to demonstrate clinical significance. The statistical analysis for OR and 95% Confidence Interval (CI) were calculated using the Prism Software

CR, complete response; RP, partial response; SD, stable disease; and PD, progressive disease.

### CERK overexpression promotes TNBC growth and migration, and alleviates toxicity-induced by chemotherapy

**Fig. 2:**
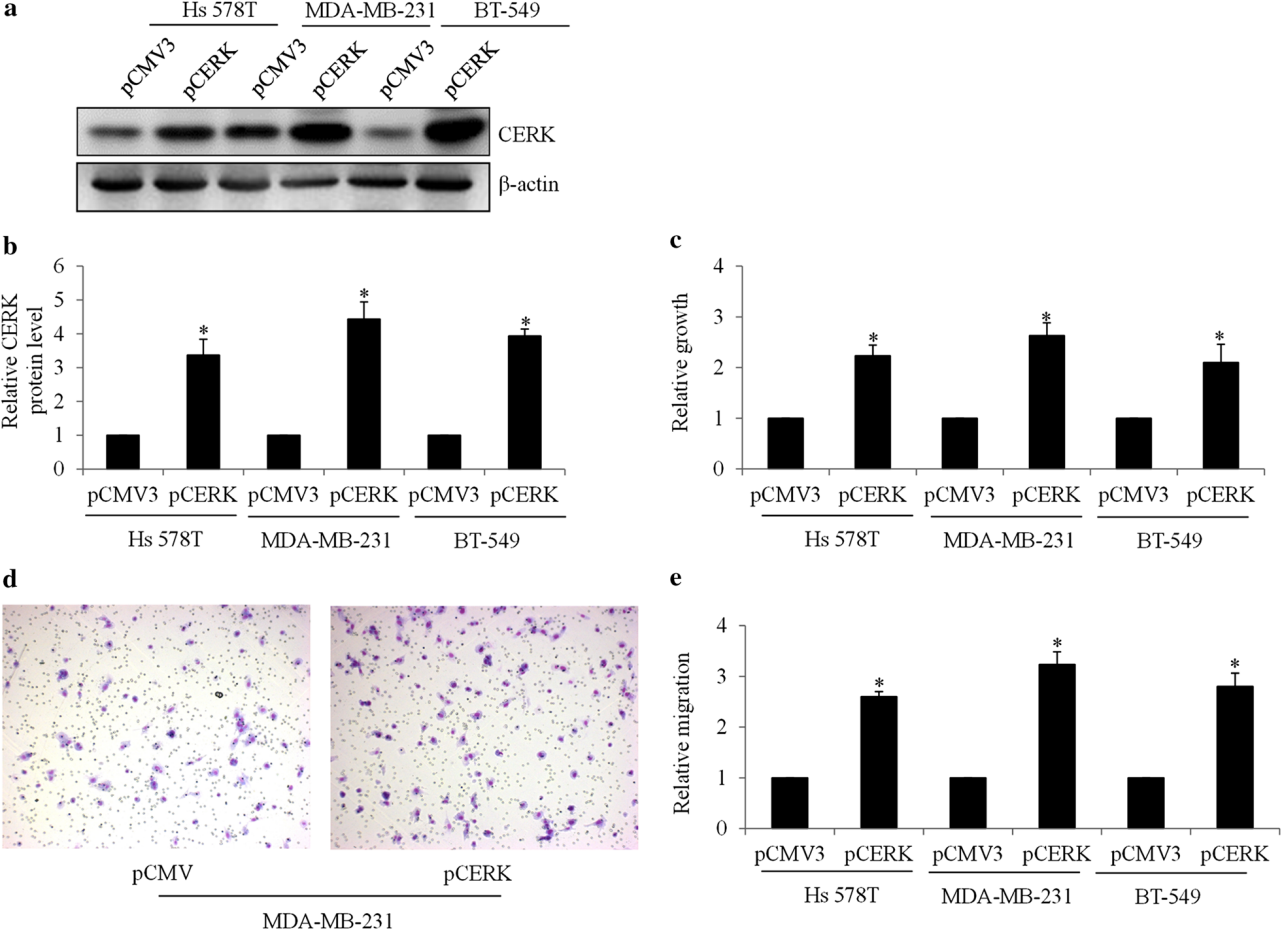
CERK overexpression promotes TNBC cell growth and migration. **a** Western blot image of CERK in TNBC cells after transfecting cells with pCMV3 and pCERK. Overexpression of CERK increases CERK level (**b**) and proliferation (**c**) in TNBC cell lines: Hs 578T, MDA-MB-231, and BT-549. **d** Representative images of Boyden chamber assay showing migrated MDA-MB-231 cells overexpressing CERK. **e** Quantification shows a significant increase in the number of migrated cells in TNBC cell lines after CERK overexpression. Proliferation and migration were measured after 72- and 24-h incubation; *P < 0.05, compared with pCMV3

**Fig. 3:**
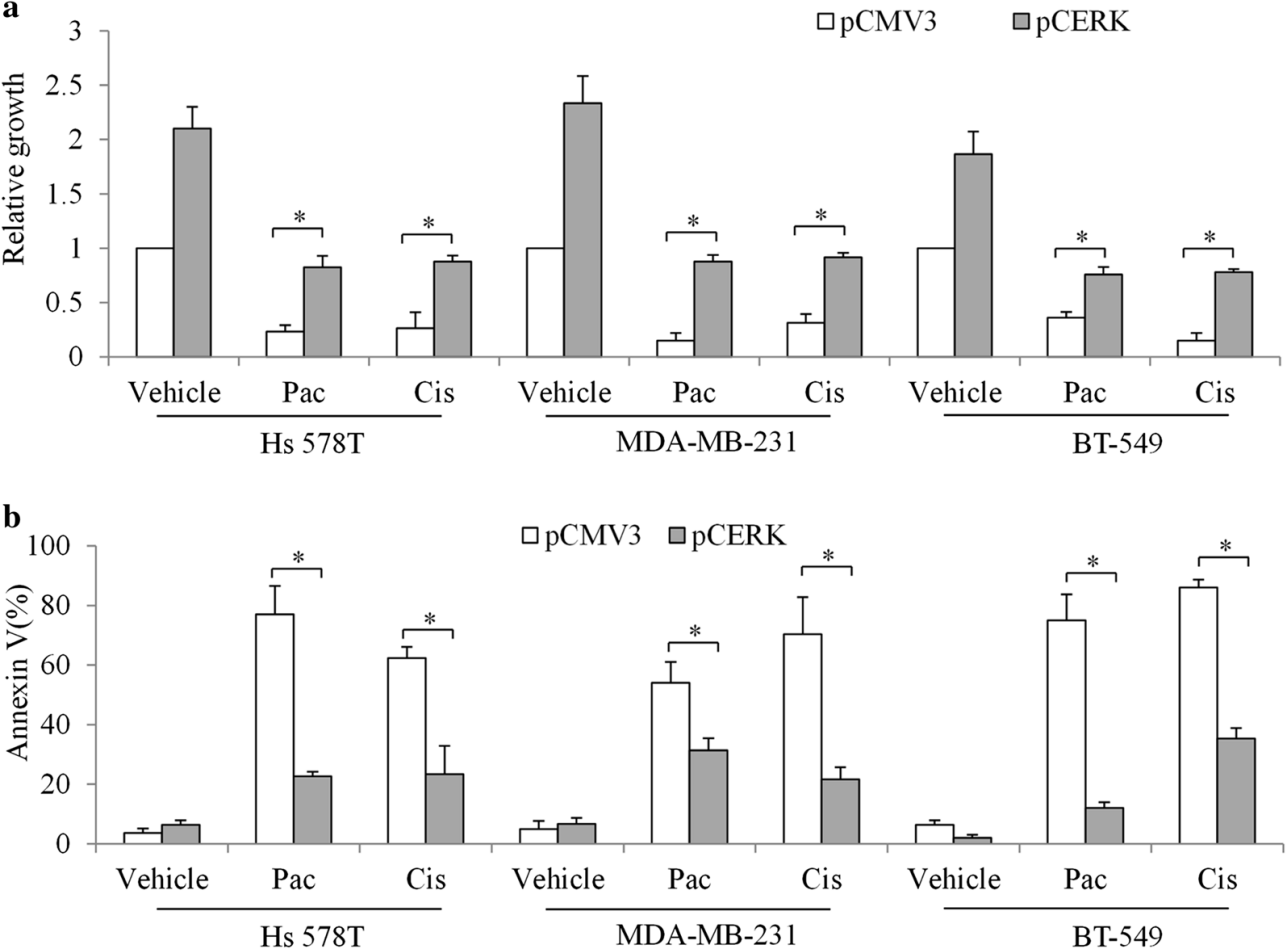
CERK overexpression alleviates cytotoxicity of chemotherapeutic agents in TNBC cells. CERK overexpression alleviates the anti-proliferative (**a**) and pro-apoptotic (**b**) effects of paclitaxel and cisplatin in TNBC cells. Paclitaxel at 2 µM and cisplatin at 50 µM were used in proliferation and apoptosis assays. Proliferation and migration were measured after 72-h incubation; *P < 0.05, compared with pCMV3

To understand the role of CERK in TNBC, we performed gain-of-function analysis by overexpressing CERK in TNBC cells. Multiple cell lines, such as MD-MBA-231, Hs 578T, and BT-549, which are commonly used and representative of an in vitro TNBC model [18], were used in our study. We found that overexpression of CERK resulted in increased levels of CERK by three- to four-fold in TNBC cells (Fig. 2a, b). In addition, we observed a significant increase in growth and migration in CERK-overexpressing TNBC cells (Fig. 2c–e). Specifically, CERK overexpression led to a ~ three-fold increase in growth and migration. We next exposed CERK-overexpressing cells to first-line chemotherapeutic agents for TNBC and assessed cell growth and survival. We found that CERK overexpression alleviated the anti-proliferative and pro-apoptotic effects of paclitaxel in TNBC cells (Fig. 3). Of note, the reduced cytotoxicity due to CERK overexpression was not limited to paclitaxel: the inhibitory effects of cisplatin in TNBC cells were also alleviated. The observations are specific to CERK overexpression, because transfection alone did not affect TNBC cell growth, migration, and survival (Additional file 1: Fig. S1). Our results obtained from multiple cell lines suggest a general role for CERK in contributing to growth, survival, and migration and protecting TNBC cells from chemo-induced toxicity. In addition, in CERK-overexpressing cells, chemotherapeutic drugs significantly reversed the stimulatory effect of CERK overexpression on TNBC cell growth (Fig. 3a). In addition, chemotherapeutic drugs significantly increased apoptosis in CERK-overexpressing cells (Fig. 3b). These demonstrate that chemotherapy is still effective in inhibiting proliferation and inducing apoptosis in CERK-overexpressing cells.

### CERK inhibition supresses TNBC and augments efficacy of chemotherapy

**Fig. 4:**
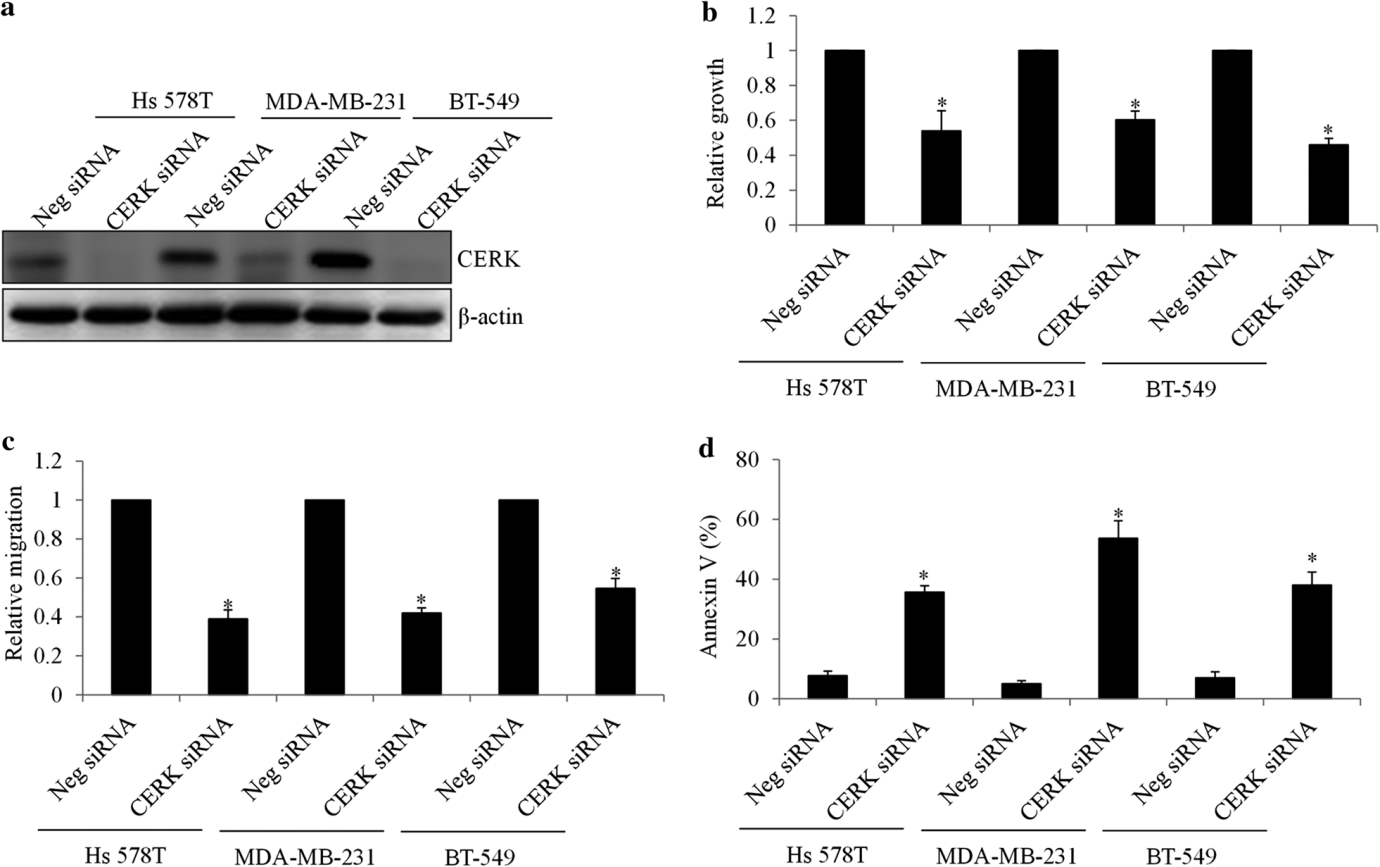
CERK knockdown suppresses TNBC growth, migration, and survival. **a** Representative western blot image showing CERK level in TNBC cells after siRNA knockdown. CERK siRNA knockdown significantly inhibits growth (**b**) and migration (**c**), and induces apoptosis (**d**) in TNBC cells. *P < 0.05, compared with Neg siRNA

**Fig. 5:**
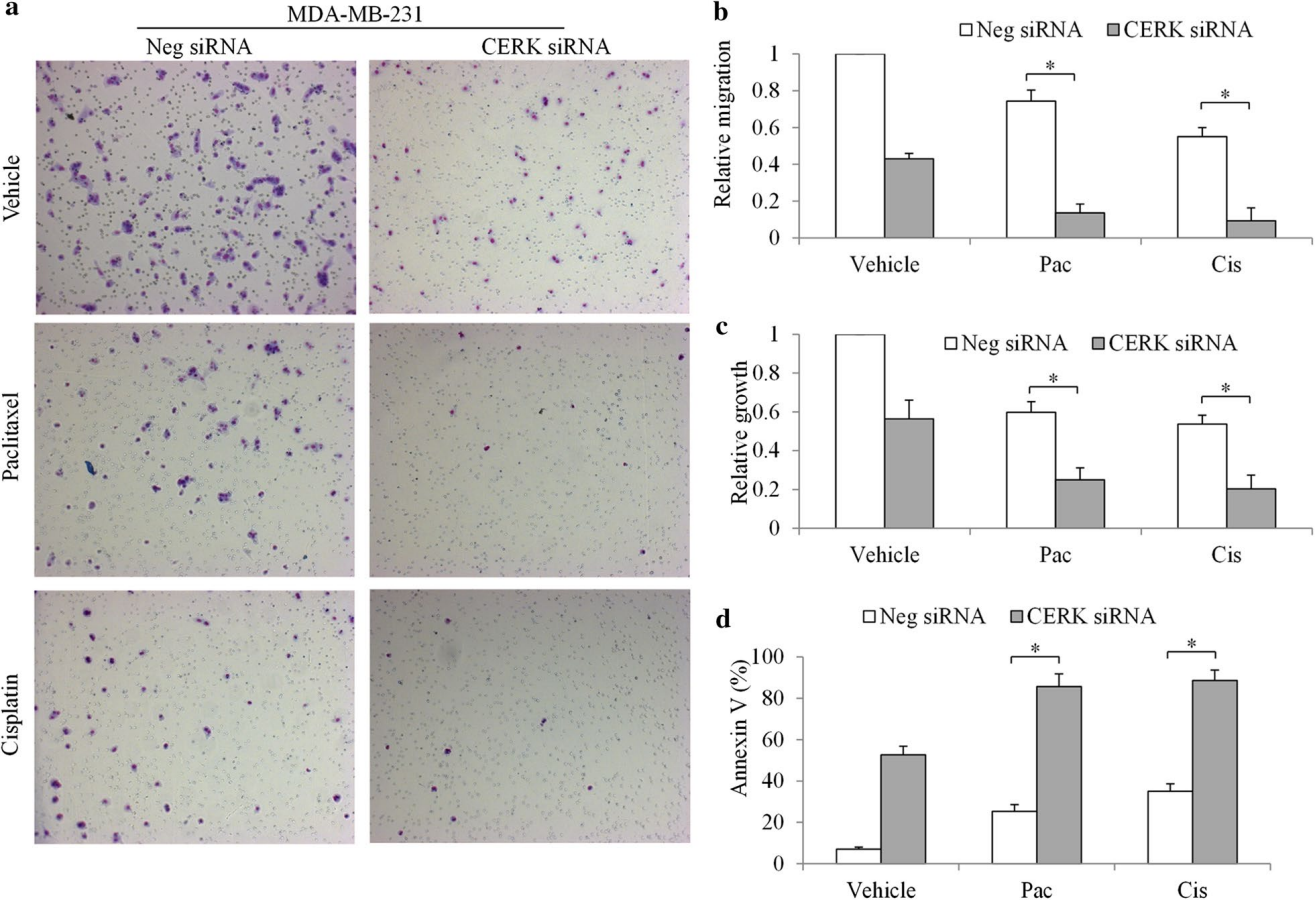
CERK knockdown augments cytotoxicity of chemotherapeutic agents in TNBC cells. **a** Representative images of Boyden chamber assay showing migrated cells among the CERK-depleted MDA-MB-231 cell population in the absence and presence of paclitaxel or cisplatin. CERK depletion significantly augments the anti-migratory (**b**), anti-proliferative (**c**), and pro-apoptotic (**d**) effects of cisplatin and paclitaxel. Paclitaxel (at 100 nM) and cisplatin (at 1 µM) were added to the cell medium at 24 h post-transfection; *P < 0.05, compared with the chemotherapeutic agent alone

We next validated the role of TNBC using the loss-of-function approach via siRNA knockdown with two siRNAs targeting different region of CERK. Western blot indicated minimal CERK protein levels in TNBC cells transfected with CERK siRNA (Fig. 4a). We further observed decreased growth and migration, and increased apoptosis, in CERK-depleted TNBC cells (Fig. 4b–d and Additional file 1: Fig. S2). In particular, CERK depletion inhibited growth by ~ 50%, migration by ~ 60%, and induced apoptosis by ~ 50%. In addition, a further reduction of migration and growth, and a further induction of apoptosis, were observed in CERK-depleted cells in the presence of cisplatin or paclitaxel compared with those subjects to drug alone or CERK inhibition alone (Fig. 5 and Additional file 1: Figs. S2 and S3). These results demonstrate that CERK inhibition suppresses TNBC and significantly augments the efficacy of chemotherapy. In CERK-depleted cells, the addition of paclitaxel or cisplatin resulted in further inhibition of growth and migration, and a further increase in apoptosis (Fig. 5), suggesting that chemotherapeutic drugs augment the inhibitory effects of CERK depletion.

### CERK mediates resistance in TNBC by regulating multiple oncogenic pathways

**Fig. 6:**
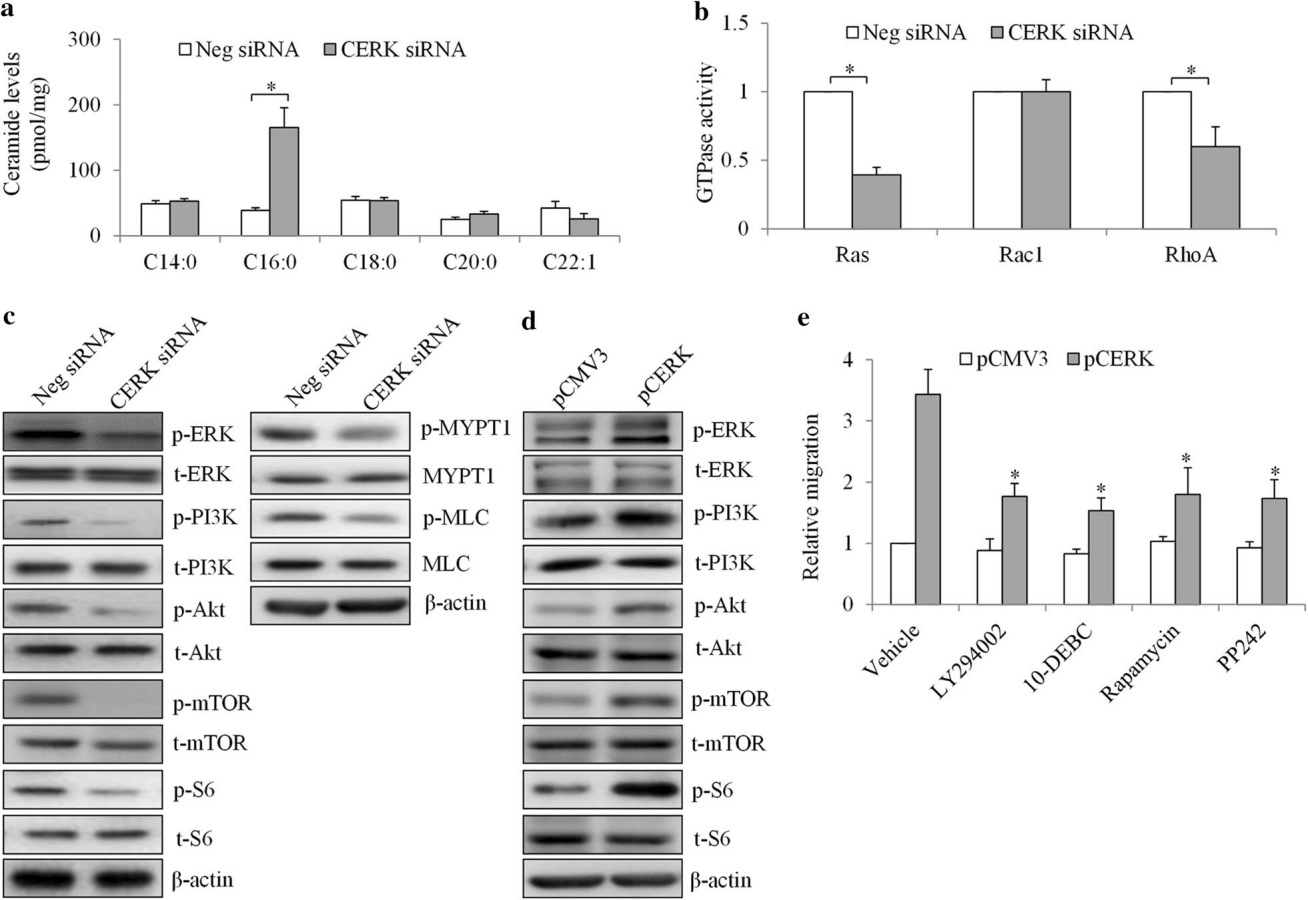
CERK positive regulatory effects are mediated by Ras/Mek, PI3K/Akt/mTOR, and Rho signaling in TNBC cells. **a** CERK depletion significantly increases the C16:0 level in TNBC cells. **b** CERK depletion significantly decreases the activity of Ras and RhoA, but not that of Rac1, in TNBC cells. **c** Representative western blot images showing the levels of phosphorylated molecules involved in Ras/Mek, PI3K/Akt/mTOR, and Rho signaling pathways after CERK depletion. **d** Representative western blot images showing the levels of phosphorylated molecules involved in Ras/Mek and PI3K/Akt/mTOR pathways in cells after CERK overexpression. **e** LY294002 (5 µM), 10-DEBC (1 µM), Rapamycin (5 µM), and PP242 (1 µM) significantly reverse the increased migration in CERK-overexpressing cells. Western blot analysis, ceramide measurement, and small GTPase activity analysis were performed after 24-hour incubation; *P < 0.05, compared with the Neg siRNA or Vehicle

Sphingomyelin metabolism has been shown to mediate cancer growth, survival, drug resistance, and host angiogenesis by modulating oncogenic signaling, such as Ras signal transmission [19, 20]. Given the known role of CERK in regulating ceramide levels [5], we first determined the ceramide level in TNBC cells after CERK inhibition. We found that CERK knockdown significantly increased ceramide levels, particularly of C16: 0 (Fig. 6a). CERK depletion resulted in a significant reduction in Ras and RhoA activity, whereas Rac1 activity was not affected in TNBC cells (Fig. 6b). Consistent with the inhibition of Ras activity, we found that CERK depletion decreased phosphorylation of ERK, which acts downstream of Ras (Fig. 6c). In addition, decreased phosphorylation of PI3K, Akt, mTOR, and S6 were observed in CERK-depleted cells, demonstrating the suppression of the PI3K/Akt/mTOR pathway by CERK inhibition. Phosphorylation of the myosin phosphatase-targeting subunit 1 (MYPT1) and myosin light chain (MLC), two downstream effectors of RhoA signalling [21], were decreased in CERK-depleted cells (Fig. 6c), suggesting that CERK inhibits the RhoA signalling pathway in TNBC.

In contrast to CERK knockdown, CERK overexpression increased phosphorylation of ERK, PI3K, Akt, mTOR, and S6 in TNBC cells (Fig. 6d). To confirm that the role of CERK is mediated by the PI3K/Akt/mTOR pathway, we performed rescue experiments using specific inhibitors of PI3K (e.g., LY294002), Akt (e.g., 10-DEB) and mTOR (e.g., rapamycin and PP242) at concentrations that lead to specific inhibition without off-target effects [8, 22]. The results showed that the inhibitors of these kinases partially, but significantly, reversed the pro-migratory effect of CERK overexpression (Fig. 6e), suggesting that PI3K/Akt/mTOR is required for the role of CERK in mediating TNBC cell migration. We performed comparison analysis on the migration of pCMV3 and pCERK cells in the absence or presence of inhibitors. We found that there was no difference in migration between pCMV3 cells treated with vehicle or pathway inhibitors, whereas there was a significant difference between pCERK cells treated with vehicle or pathway inhibitors (Fig. 6e). These results demonstrate that the pathways shown in our study are specific to CERK overexpression.

## Discussion

Our findings suggest that although TNBC is a highly heterogeneous malignant tumor at the molecular and cellular levels, driver genes can modulate the responses of TNBC to drug treatment and influence clinical outcome. We show how CERK contributes to the heterogeneity of responses seen among TNBC treated with chemotherapeutic agents. Of note, our findings also highlight CERK as a sensitizing therapeutic target to overcome TNBC chemoresistance. To our knowledge, this is the first report of CERK as a biomarker linking to clinical outcome of chemotherapy in TNBC.

CERK expression varies significantly in both tumor and adjacent breast tissues in TNBC patients (Fig. 1a, b), which is consistent with the RNA-seq data for CERK in normal and tumor breast samples from The Cancer Genome Atlas. It is interesting to note that the upregulation of CERK expression (tumor/normal > 2) is found only in some TNBC patients, with a significant association with chemoresistance (Table 1). Previous studies using cell models and publicly available microarray data show that elevated CERK expression is linked with metastatic breast cancer and increased risk of recurrence in patients with breast cancer [12, 13, 23]. Specifically, CERK overexpression is also associated with decreased recurrence-free survival in patients with ER-negative breast cancer [14]. Our work agrees with and further extends these previous studies by demonstrating that CERK upregulation is associated with chemoresistance in patients with TNBC, the most aggressive subtype of breast cancer. However, whether its association with chemoresistance and prognostic impact depends on aggressive prognostic factors is inconclusive. Prospective studies are necessary to validate the clinical impact of CERK in refining risk stratification and response to specific therapeutic modalities in breast cancer.

By elucidating the effects of CERK upregulation, we demonstrate that CERK alleviates the efficacy of paclitaxel and cisplatin in TNBC (Figs. 3 and 5). Thus, in showing that chemoresistance is caused by CERK upregulation, we further demonstrated that CERK inhibition sensitized TNBC cells to chemotherapeutic drugs. Our findings support the increasingly recognized cytoprotective role of CERK in a variety of stress conditions, such as UV irradiation and serum starvation, and provide a new example of chemoresistance [24, 25]. Apart from chemoresistance, we showed that CERK promoted TNBC migration (Fig. 2d, e), which is in line with the findings of a recent study on the contribution of CERK to migration and invasion in metastatic breast cancer cells [13]. Similar to CERK, C1P was reported to increase migration and invasion in pancreatic cancer cells [8]. In contrast, CERK was also reported to negatively regulate lamellipodium formation, migration, and metastasis of A549 lung cancer cells [26]. Taken together, these findings suggest that the regulatory role of CERK in migration might be cancer-cell type specific.

The mechanisms underlying the role of CERK role in cancer are still largely unknown. CERK was shown to be upregulated following HER2/neu pathway inhibition in breast cancer cells [12]. In metastatic breast cancer cells, PI3K, Akt, mTOR, and RhoA were demonstrated to be responsible for migration regulation by CERK [13]. Using both knockdown and overexpression approaches, our study demonstrated that, in addition to PI3K/Akt/mTOR and Rho pathways, CERK activates the Ras/ERK pathway in TNBC cells (Fig. 6b–e). The activation of multiple oncogenic pathways as a result of CERK overexpression correlates well with the positive regulatory roles of CERK in TNBC and other types of breast cancer cells. Because CERK is responsible for the balance of ceramide and C1P via the phosphorylation of ceramide to produce C1P, we expected to observe ceramide accumulation by CERK inhibition: this was indeed confirmed in the setting of CERK-depleted TNBC cells (Fig. 6a). The accumulation of ceramide might explain the anti-proliferative and pro-apoptotic effects of CERK inhibition in TNBC cells. Signal transmission of small GTPases, such as Ras, is modulated by plasma membrane lipids due to their dependence on spatial clustering of lipids [19]. Our work is the first to demonstrate that CERK inhibition suppresses Ras/ERK signaling, mostly likely via the regulation of lipid metabolism. Although infrequent canonical mutations occur in Ras/ERK, this pathway is activated after chemotherapy in TNBC [27]. Our study highlights the biological implication of CERK for Ras/ERK pathway activity in TNBC.

## Conclusions

Although our data focus on the effect of CERK upregulation on chemotherapeutic responses in TNBC, it is possible that CERK also accounts for treatment resistance among other subtypes of breast cancer or even other cancers. In the case of chemoresistance in individuals with CERK upregulation, pharmacological inhibitors of CERK could be used as an adjuvant therapy in TNBC. Our work also provides a mechanistic understanding of how CERK contributes TNBC chemoresistance.

## Supplementary Information

**Additional file 1: Fig. S1**. Transfection alone does not affect TNBC cell growth, migration and survival. Proliferation (A), migration (B) and apoptosis (C) of TNBC parental cells and cells transfected with Neg siRNA or pCMV3. **Fig. S2.** CERK knockdown augments cytotoxicity of chemotherapeutic agents in TNBC cells. CERK depletion significantly augments the anti-migratory (A), anti-proliferative (B) and pro-apoptotic (C) effects of cisplatin and paclitaxel in BT-549 and Hs 578T cells. Paclitaxel at 100 nM and cisplatin at 1 μM were added to the cell medium at 24 h post-transfection. *P < 0.05, compared to chemotherapeutic agent alone. **Fig. S3.** CERK knockdown augments cytotoxicity of chemotherapeutic agents in TNBC cells. CERK depletion using another siRNA (CERK siRNA#2) significantly augments the anti-proliferative (A to C), anti-migratory (D to F) and pro-apoptotic (G to I) effects of cisplatin and paclitaxel in MDA-MB-231, BT-549 and Hs 578T cells. Paclitaxel at 100 nM and cisplatin at 1 μM were added to the cell medium at 24 h post-transfection. *P < 0.05, compared to chemotherapeutic agent alone.

## Data Availability

Data and materials are available upon request.
