# Supplementary material for: Ceramide kinase mediates intrinsic resistance and inferior response to chemotherapy in triple‐negative breast cancer by upregulating Ras/ERK and PI3K/Akt pathways
Source: Cancer Cell Int. 2021 Jan 11;21:42. doi: 10.1186/s12935-020-01735-5 (PMC7802356; doi:10.1186/s12935-020-01735-5)
Supplement: Supplementary file 1 — Additional file 1: Fig. S1. Transfection alone does not affect TNBC cell growth, migration and survival. Proliferation (A), migration (B) and apoptosis (C) of TNBC parental cells and cells transfected with Neg siRNA or pCMV3. Fig. S2. CERK knockdown augments cytotoxicity of chemotherapeutic agents in TNBC cells. CERK depletion significantly augments the anti-migratory (A), anti-proliferative (B) and pro-apoptotic (C) effects of cisplatin and paclitaxel in BT-549 and Hs 578T cells. Paclitaxel at 100 nM and cisplatin at 1 μM were added to the cell medium at 24 h post-transfection. *P < 0.05, compared to chemotherapeutic agent alone. Fig. S3. CERK knockdown augments cytotoxicity of chemotherapeutic agents in TNBC cells. CERK depletion using another siRNA (CERK siRNA#2) significantly augments the anti-proliferative (A to C), anti-migratory (D to F) and pro-apoptotic (G to I) effects of cisplatin and paclitaxel in MDA-MB-231, BT-549 and Hs 578T cells. Paclitaxel at 100 nM and cisplatin at 1 μM were added to the cell medium at 24 h post-transfection. *P < 0.05, compared to chemotherapeutic agent alone. [file 12935_2020_1735_MOESM1_ESM.doc]

**Ceramide kinase mediates intrinsic resistance and inferior response to chemotherapy in triple negative breast cancer via regulating multiple oncogenic pathways**

**Fig. S1: Transfection alone does not affect TNBC cell growth, migration and survival.** Proliferation (A), migration (B) and apoptosis (C) of TNBC parental cells and cells transfected with Neg siRNA or pCMV3.

**Fig. S2: CERK knockdown augments cytotoxicity of chemotherapeutic agents in TNBC cells.** CERK depletion significantly augments the anti-migratory (A), anti-proliferative (B) and pro-apoptotic (C) effects of cisplatin and paclitaxel in BT-549 and Hs 578T cells. Paclitaxel at 100 nM and cisplatin at 1 μM were added to the cell medium at 24 h post-transfection. *p<0.05, compared to chemotherapeutic agent alone.

**Fig. S3: CERK knockdown augments cytotoxicity of chemotherapeutic agents in TNBC cells.** CERK depletion using another siRNA (CERK siRNA#2) significantly augments the anti-proliferative (A to C), anti-migratory (D to F) and pro-apoptotic (G to I) effects of cisplatin and paclitaxel in MDA-MB-231, BT-549 and Hs 578T cells. Paclitaxel at 100 nM and cisplatin at 1 μM were added to the cell medium at 24 h post-transfection. *p<0.05, compared to chemotherapeutic agent alone.
